# Supplementary material for: Far-travelled 3700 km lateral magma propagation just below the surface of Venus
Source: Nat Commun. 2024 Feb 26;15:1759. doi: 10.1038/s41467-024-45603-6 (PMC10897134; doi:10.1038/s41467-024-45603-6)
Supplement: Supplementary file 2 — Description of Additional Supplementary Files [file 41467_2024_45603_MOESM2_ESM.pdf]

Description of Additional Supplementary Files

File Name: Supplementary Data 1
Description: Shapefiles shown in this manuscript (Figure 1), including the generalized linework for the Ozza Mons radiating graben system and the detailed tracing of the Great Dyke of Atla Regio (GDAR).
